# ﻿Two new species of *Cora* (lichenized Basidiomycota, Lichenomphaliaceae) and additional records from Bolivia

**DOI:** 10.3897/mycokeys.126.165395

**Published:** 2025-12-02

**Authors:** Magdalena Oset, Adam Flakus, Beata Guzow-Krzemińska

**Affiliations:** 1 Department of Plant Taxonomy and Nature Conservation, University of Gdańsk, Wita Stwosza 59, PL-80-308 Gdańsk, Poland University of Gdańsk Gdańsk Poland; 2 W. Szafer Institute of Botany, Polish Academy of Sciences, Lubicz 46, PL-31-512 Krakow, Poland W. Szafer Institute of Botany, Polish Academy of Sciences Krakow Poland

**Keywords:** Basidiolichens, cyanolichens, molecular barcoding, neotropics, new species

## Abstract

Two new species of lichens, *Cora neoparabovei* Oset, Flakus & Guzow-Krzem. and *C. neopseudobovei* Oset, Flakus & Guzow-Krzem., are described based on material collected in Bolivia. The study is based on morphological and anatomical examinations, molecular phylogenetic analysis and haplotype network analysis of the nuITS rDNA sequences. Phenotypically, *Cora neoparabovei* is characterised by the long, concentrically arranged setae on the surface of the thallus and densely pilose margins, whereas in *C. neopseudobovei* the diagnostic features are the brown upper surface thallus when fresh, pale yellow to orange-yellow when dry, with slightly visible concentric colour zonation when dry and also with involute, creamy white margins. In addition, three species, i.e., *Cora arcabucana* B. Moncada, C. Rodr. & Lücking, *C. cuzcoensis* Holgado, Rivas Plata & Perlmutter, and *C. undulata* L.Y. Vargas, B. Moncada & Lücking, are reported as new to Bolivia. New records of *C. aspera* Wilk, Lücking & E. Morales from Bolivia are reported.

## ﻿Introduction

According to Lücking et al. (2014c) lichenized Basidiomycota make up less than one percent of all lichenized fungi in terms of species number. However, in ecosystems and habitats such as tropical alpine regions and conserved forests, lichenized Basidiomycota play an important role as a component of tropical forests (see also Petersen 1967; Oberwinkler 1970, 1984, 2012; Gómez 1972; Parmasto 1978; Lutzoni and Vilgalys 1995; Redhead et al. 2002; Lawrey et al. 2007, 2009; Ertz et al. 2008; Yánez et al. 2012). Within Basidiomycota, the family Lichenomphaliaceae (Lücking & Redhead) Vizzini, Consiglio & P. Alvarado is represented by eight genera: *Acantholichen* P.M. Jørg., *Arrhenia* Fr., *Cora* Fr., *Corella* Vain., *Cyphellostereum* D.A. Reid, *Dictyonema* C. Agardh ex Kunth, *Eonema* Redhead, Lücking & Lawrey, and *Lichenomphalia* Redhead, Lutzoni, Moncalvo & Vilgalys (Vizzini et al. 2024). The most diverse genus within Lichenomphaliaceae is *Cora*, with over 100 species formally described using a combination of morphology, anatomy, habitat ecology, and nuITS rDNA barcoding, but many still await formal recognition as 450 species are predicted to exist (Lücking et al. 2013, 2014b, 2014c, 2014d, 2015, 2017, 2020; Dal Forno et al. 2019).

*Cora* is characterised by foliose to macrosquamulose thalli forming distinct layers (cortex, photobiont layer, medulla) and producing corticoid basidiocarps on the lobe underside (see Dal Forno et al. 2013). In many lichen groups taxonomy can be based on morphological, anatomical or chemical characters. However, in the case of the genus *Cora*, traditional revisions based largely on herbarium collections failed to recognise important field characters (e.g. lobe arrangement, colour, and substrate or hymenophore anatomy) to establish species which led to numerous misclassifications of taxa. These traits are not widely used for diagnostic purposes due to the excessive plasticity of the thallus and changes in morphology resulting, from among other things, habitat conditions or variations in these traits due to changes in thallus hydration. This makes species within this genus much more difficult to distinguish. According to Dal Forno et al. (2022), it was the main reason why historically only a single foliose species *Cora glabrata* (Spreng.) Fr. (previously named *Dictyonema pavonium* (Sw.) Parmasto and subsequently *D. glabratum* (Spreng.) D. Hawksw. was recognised. According to Dal Forno et al. (2022) the best method for species identification in the genus *Cora* is DNA barcoding using nuITS rDNA. In the genus *Cora* it has been generally found that the topology of phylogeny based on ITS is highly consistent with the topology of other markers, such as nuLSU and RPB2, suggesting that ITS accurately distinguishes these lineages (Dal Forno et al. 2022). Therefore, for *Cora* taxonomy, DNA barcoding is the best and most efficient research method, which has already significantly increased the number of taxa described. This conclusion is also supported by this study.

Despite the intensification of research on the genus *Cora* in Neotropical areas (Dal Forno et al. 2019, 2022; Lücking et al.2013, 2014b, 2014c, 2014d, 2015, 2017, 2020), there are still areas where this genus has not been thoroughly investigated. One such country is Bolivia, which is one of the two landlocked South American countries located in the central part of the continent. Bolivia is the continent’s highest country in terms of average altitude, with more than half of the country’s area made up of highlands and mountains. Also, the country is characterized by tropical and, in many places, dry climates. According to Ibisch and Mérida (2004) and Josse et al. (2003), twelve ecoregions and twenty-three sub-regions are present in Bolivia. All together these make it very diverse in terms of biodiversity and the country is ranked among the ones with the highest biodiversity in the world (e.g. Ibisch and Mérida 2004; Josse et al. 2003; Rodriguez-Flakus et al. 2016).

Biodiversity studies in Bolivia remain rather poorly explored, and lichens are no exception. The late development of lichenological research in Bolivia meant that by 1998, only 150 lichen species had been recorded. As a result of surveys conducted in subsequent years, this number has increased to about 2,000 species. This suggests that only about half of Bolivia’s lichen biota has been documented so far, and many additional new taxa are undoubtedly yet to be discovered there (Rodriguez-Flakus et al. 2016 and literature cited therein). To date, 18 species of the genus *Cora* have been recorded from Bolivia (e.g. Flakus and Lücking 2008; Lücking et al. 2013, 2014c, 2017; Ertz et al. 2015; Flakus et al. 2016; Rodriguez-Flakus et al. 2016 and lit. cited therein; Guzow-Krzemińska et al. 2019; Oset et al. 2024). Recently, modern approaches in *Cora* taxonomy, including phylogenetic analyses, have been applied to Bolivian collections, resulting in the recording of additional species, including taxa new to science.

This paper is a further contribution and presents the descriptions and records of five additional *Cora* species, including two new to science (*C. neoparabovei* and *C. neopseudobovei*), three new to Bolivia (*C. arcabucana* B. Moncada, C. Rodr. & Lücking, *C. cuzcoensis* Holgado, Rivas Plata & Perlmutter, and *C. undulata* L.Y. Vargas, B. Moncada & Lücking), and new records of *C. aspera* Wilk, Lücking & E. Morales.

## ﻿Materials and methods

### ﻿Taxon sampling

*Cora* specimens were collected in the Yungas and Tucumano-Boliviano regions during fieldwork carried out between 2010 and 2014. The specimens are deposited at KRAM, LPB, and UGDA herbaria. Morphology and anatomy were examined using Nikon SMZ800N dissecting microscope and ZEISS Axioskop compound microscope. Character assessment was based on the morphological and anatomical traits from *Cora* described by Lücking et al. (2013, 2014a, 2017) and Vargas et al. (2014). Secondary compounds were analysed using thin-layer chromatography (TLC) in solvents A and C (Orange et al. 2001).

### ﻿DNA extraction, PCR amplification and sequencing

A total of 18 new specimens of *Cora* from Bolivia were used for the molecular study. The new nuITS rDNA sequences were compared with *Cora* sequences available in the GenBank database using BLAST search (Altschul et al. 1997). The nuITS rDNA sequences of representative taxa belonging to the genus *Cora*, based on the analyses of Dal-Forno et al. (2022) and Oset et al. (2024), were aligned with new sequences. *Corella tomentosa* Vain. (KJ780617) and *C. zahlbruckneri* Schiffn. (KJ780592) were used as an outgroup (Suppl. material 1). For new DNA extractions from Bolivian material, two separate thallus fragments were taken from each specimen to allow cross-checking of the results and account for potential sample contamination.

Total genomic DNA was extracted using the Plant & Fungi DNA Purification Kit (Eurx, Poland), following the manufacturer’s protocol and modified CTAB method (Guzow-Krzemińska and Węgrzyn 2000). Fungal nuITS rDNA was amplified using the primers ITS5 and ITS4 (White et al. 1990). The same primers were used for sequencing. For PCR amplification, Start-Warm HS-PCR Mix (A&A Biotechnology, Poland) was used with the following parameters: an initial denaturation at 94 °C for 3 min and 33 cycles of denaturation at 94 °C for 30 sec; annealing at 52° for 45 sec; and extension at 72 °C for 1 min followed by a final extension at 72 °C for 10 min. The PCR products were purified with the Clean-Up Kit (A&A Biotechnology, Poland) according to the manufacturer’s instructions. Sequencing was performed in Macrogen (the Netherlands) (http://www.macrogen.com).

### ﻿Sequence editing and alignments

Forward and reverse sequences of the nuITS rDNA region were assembled using BIOEDIT v. 7.25. (Hall 1999). The assembled sequences were analysed using BLAST search (Altschul et al. 1997) at NCBI (https://www.ncbi.nlm.nih.gov). Sequences used for the phylogenetic analyses were selected based on Dal-Forno et al. (2022) and Oset et al. (2024) and are presented in Suppl. material 1 together with GenBank accession numbers, voucher numbers, country of origin, and source publication. A single sequence was used for most species, with multiple sequences used for species that are newly recorded. The final alignment of representatives of *Cora* spp. was generated in MAFFT using auto option and default parameters (Kuraku et al. 2013; Katoh et al. 2019). The alignment was trimmed using MEGA-11 (Tamura et al. 2021). The final alignment consisted of 109 sequences and 840 sites.

A second alignment of newly sequenced samples together with sequences of reference taxa obtained from GenBank was made in Seaview software (Galtier et al. 1996; Gouy et al. 2010) employing the muscle option. The alignment included only the closely related species: *C. bovei*, *C. palaeotropica*, *C. parabovei*, *C. pseudobovei*, *C. neoparabovei*, *C. neopseudobovei*, *Cora* sp., and consisted of 20 sequences and 752 sites.

### ﻿Network and phylogenetic analyses

IQ-TREE analysis was performed to find the best-fitting nucleotide substitution model (Nguyen et al. 2015) with the model selection restricted to models implemented in MrBayes. TIMe+I+G4 was chosen based on BIC. The search for maximum likelihood tree with 100 bootstrap replicates was performed using IQ-TREE (Nguyen et al. 2015) on the CIPRES Web Portal (Miller et al. 2010).

Bayesian analysis was carried out using a Markov Chain Monte Carlo (MCMC) method, in MrBayes v. 3.2.6 (Huelsenbeck and Ronquist 2001; Ronquist and Huelsenbeck 2003) on the CIPRES Web Portal (Miller et al. 2010) using a previously selected model. Two parallel MCMC runs were performed, each using four independent chains and ten million generations, sampling every 1000^th^ tree. The resulting log files were analysed using Tracer 1.7.2 (Rambaut et al. 2018). Posterior probabilities (PP) were determined by calculating a majority-rule consensus tree after discarding the initial 25% trees of each chain as the burn-in. The convergence of the chains was confirmed by the convergent diagnostic of the Potential Scale Reduction Factor (PSRF), which approached 1, and the ‘average standard deviation of split frequencies’ was < 0.01 (Ronquist et al. 2005).

Phylogenetic trees were visualised using FigTree v. 1.4.3 (Rambaut 2009) and modified in Inkscape (https://inkscape.org/). Bootstrap support (BS values ≥ 70) and PP values (values ≥ 0.95) are given near the branches on the phylogenetic tree.

PopART software (https://popart.maths.otago.ac.nz) employing the TCS network option was used for haplotype network analyses (Clement et al. 2002). This analysis is supplemented with a table showing variable positions in the alignment of nuITS rDNA marker of *Cora bovei*, *C. neoparabovei*, *C. neopseudobovei*, *C. palaeotropica*, *C. parabovei*, *C. pseudobovei* and *Cora* sp. (Suppl. material 2) as PopArt masks sites with gaps and missing data.

## ﻿Results and discussion

**Figure 1.**
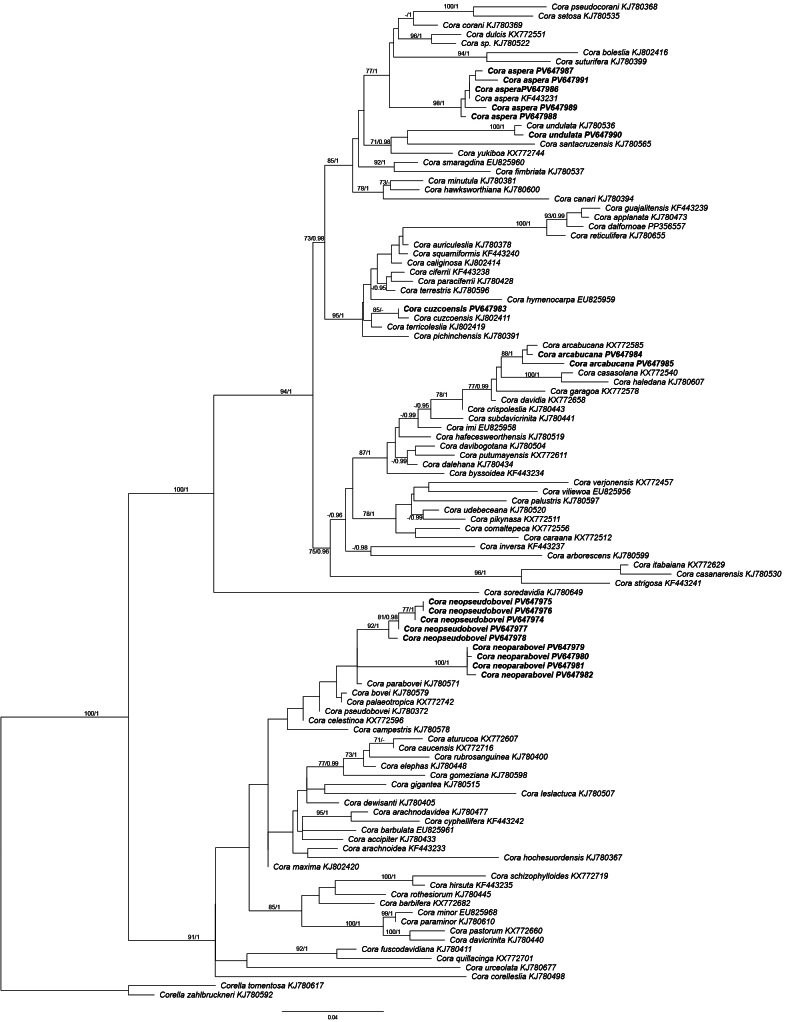
IQ-tree based on a nuITS rDNA dataset for *Cora* spp. The names of species are followed with their GenBank accession numbers (see Suppl. material 1). Newly sequenced specimens are marked in bold. Bootstrap support values (%) from IQ-tree analysis and PP support from Bayesian analysis are indicated near the branches. Sequences of *Corella tomentosa*KJ780578 and *Corella zahlbruckneri*KJ780592 were used as outgroup taxa.

Eighteen new nuITS rDNA sequences were generated for this study and used in phylogenetic analysis together with reference sequences representing different species of *Cora* (Fig. 1). Nine of these were found to represent previously described *Cora* species (Lücking et al. 2013, 2017; Vargas et al. 2014). These are *C. arcabucana* (2 specimens), *C. cuzcoensis* (1 specimen), and *C. undulata* L (1 specimen), which are new to Bolivia, and five records belong to *C. aspera*, which was recorded only on three localities in Bolivia (Fig. 1).

Nine sequences form two, closely related, distinct lineages in the *Cora* tree (Fig. 1), suggesting the presence of two new species, named here as *C. neoparabovei* and *C. neopseudobovei*. These species are related to *C. parabovei* Dal-Forno, Kukwa & Lücking. Other taxa closely related to these new species are *C. bovei* Speg., *C. campestris* Dal-Forno, Eliasaro & A.A. Spielm., *C. palaeotropica* Weerakoon, Aptroot & Lücking, *C. pseudobovei* Wilk, Dal-Forno & Lücking, and *C. celestinoa* B. Moncada, Cabr.-Amaya & Lücking. All these species are characterized by terricolous thallus with more or less brown or olive thallus when fresh. Based on their morphological similarity and the tree topology, we propose that the group of all of these species should be named as the *Cora bovei* group.

**Figure 2.**
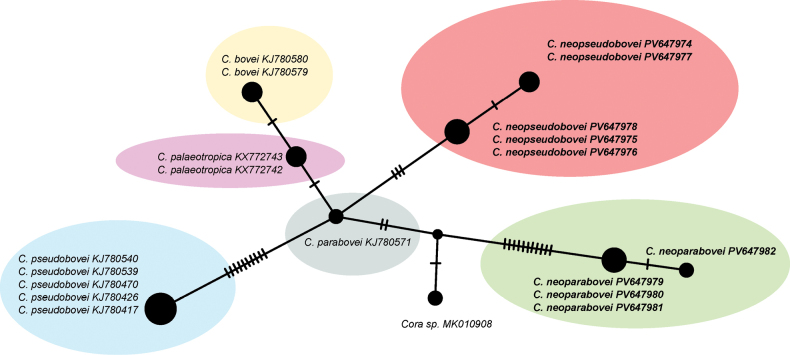
Haplotype network showing relationships between nuITS rDNA sequences from *Cora bovei*, *C. neoparabovei*, *C. neopseudobovei*, *C. palaeotropica*, *C. parabovei* and *C. pseudobovei* and *Cora* sp.. The names of species are followed with their GenBank accession numbers. The newly sequenced samples are marked in bold (see Suppl. material 1). Mutational changes are presented as hatch marks. Each species is marked in colour with ellipse.

The first novel species, *C. neoparabovei* is represented by four specimens from Bolivia. All specimens are characterised by the long, concentrically arranged setae on the surface of the thallus and densely pilose in the margins. This species is phylogenetically, morphologically and ecologically similar to *C. parabovei* and other members of the clade (Fig. 1). In the haplotype network (Fig. 2), the sequences of *C. neoparabovei* are represented by two haplotypes (but see also Suppl. material 1 and Suppl. material 2). The three specimens (PV647979, PV647980, PV647981), share the same haplotype, which differs from the second (PV647982) haplotype in a single site. These differ from *C. parabovei* (KJ780571) by thierteen or fourteen nucleotide substitutions (Fig. 2, Suppl. material 2).

The second of the new species, *C. neopseudobovei* is represented by five specimens from Bolivia. To compare the genetic diversity between the new species and other related taxa, we carried out a haplotype network analysis. In the haplotype network (Fig. 2), the sequences of *C. neopseudobovei* are represented by two haplotypes (see also Suppl. material 1 and Suppl. material 2). The sequences of two specimens (PV647974, PV647977), share the same haplotype (Fig. 2), and differ from the second haplotype (PV647975, PV647976, PV647978) in a single position. Five sequences of *C. pseudobovei* (KJ780540, KJ780539, KJ780470, KJ780426, KJ780417) have the same haplotype, which differs by thirteen or fourteen nucleotide substitutions from *C. neopseudobovei*.

It seems interesting that the new *Cora* species described here have only been found in single localities, which may presumably suggest their endemism. The occurrence of endemic species of *Cora* has been noted, for example, in the United States of America, where *Cora timucua* is endemic to Florida (Lücking et al. 2020; Dal Forno et al. 2021) and from Galapagos, including *C. galapagoensis* Dal-Forno, Bungartz & Lücking, and *C. santacruzensis* Dal-Forno, Bungartz & Yánez-Ayabaca (Dal Forno et al. 2017; Lücking et al. 2017). However, as the genus is still not well studied in many regions, some species may appear more widespread. In this paper, we report the first record of *C. arcabucana* from Bolivia, which has been known only from Colombia so far (Lücking et al. 2017), *C. cuzcoensis* previously known only from the type locality near Machu Picchu in Peru (Lücking et al. 2017), and *C. undulata*, previously noted only in Colombia (Vargas et al. 2014).

### ﻿Taxonomy

#### ﻿New species from Bolivia

##### ﻿ Cora neoparabovei

Taxon classificationFungiAgaricalesLichenomphaliaceae

Oset, Flakus & Guzow-Krzem. sp. nov.

0543095F-53A2-5F68-9C08-987ECA63FD7C

860019

Fig. 3

###### Diagnosis.

Species very similar to *C. parabovei*, but differing in the distinct phylogenetic position within the genus, and in substitution of several nucleotide positions in nuITS rDNA (see also Suppl. material 2). Taxon is characterized by long, concentrically arranged setae present on the thallus surface and densely pilose margins.

**Figure 3.**
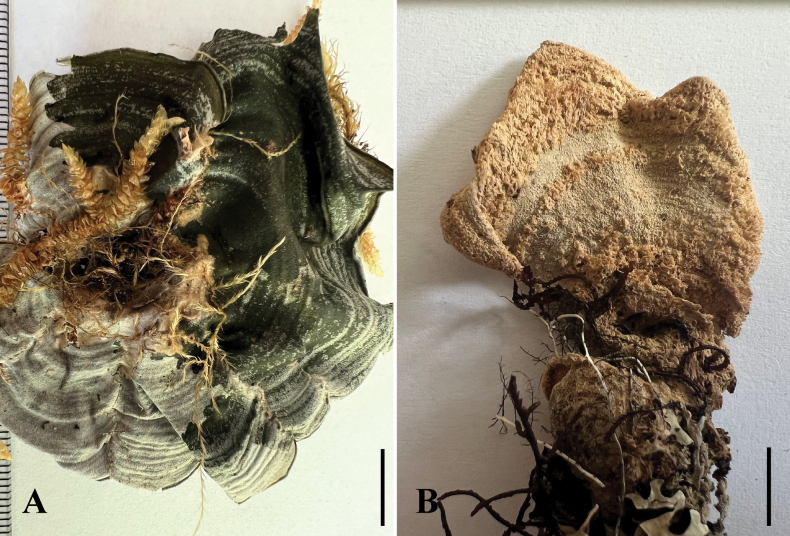
Morphology of *Cora neoparabovei* (holotype). **A.** Upper surface (on the left side thallus is dry, on the right side thallus is wet); **B.** Felty-arachnoid lower surface (thallus is dry). Scale bars: 10 mm (**A, B**).

###### Type.

Bolivia • Dept. La Paz, Prov. Nor Yungas, PNANMI Cotapata, N of Unduavi, by Sillu Tincara pre-Columbian route, 16°16'33"S, 67°52'60"W, elev. 3429 m, Yungas cloud forest, 27 June 2010, A. Flakus 16965 & P. Rodriguez (holotype KRAM; isotype LPB).

###### Description.

Thallus lichenized, terricolous over and between bryophytes, foliose, up to 8.5 cm across, composed of 3 semicircular, adjacent lobes; individual lobes up to 8 cm wide and 2.5–6.5 cm long, turned upwards, circular patches sparsely branched, without radial branching sutures, surface olive green to pale yellow in the centre with a slight concentric zonation of colour when fresh but when dried yellowish-grey. Margins thin, involute, green becoming yellow-green in the herbarium. Upper surface evenly to shallowly undulating when fresh, rugose when dry, with yellowish setae throughout, with greater density towards the centre of the thallus; setae consisting of hyphae up to 300 μm long and 5 μm wide, hyphae irregular in shape, sparingly branched and anastomosing, hyaline to very pale brown; lower surface ecorticate, felty-arachnoid (representing the exposed medulla), green-yellow when fresh, becoming yellow to brown when dried. Thallus in the section up to 550 μm thick, with upper cortex, photobiont layer, and medulla; upper cortex collapsed compacted; photobiont layer up to 250 μm thick, orange-brown (upper portion) to aerugious (lower portion); medulla 100 μm thick; clamp connections absent, papilliform hyphae absent. Hymenophore not present. No substances detected by TLC.

###### Habitat and distribution.

*Cora neoparabovei* grows on bryophytes and is known only from three localities in Nor Yungas province in La Paz department, occurring at elevations between 3210 m and 3429 m in Yungas cloud forest.

###### Etymology.

The name is derived from the Greek prefix *néos*, meaning “new”, combined with “*parabovei*”, referring to its similarity to *C. parabovei*.

###### Additional material examined.

Bolivia • Dept. La Paz; Prov. Nor Yungas, below Unduavi village, on the road La Paz – Chulumani, 16°18'27"S, 67°53'48"W, elev. 3211 m, Yungas cloud forest, on bryophytes, 31 May 2011, A. Flakus 22219 & O. Plata (KRAM L-71638; LPB). elev. 3210 m, Yungas cloud forest, on bryophytes, 31. May 2011, A. Flakus, O. Plata (UGDA–20029; LPB). • PNANMI Cotapata, 5 hours walking from Unduavi by Sillu Tincara pre-Columbian route, 16°16'33"S, 67°52'60"W, elev. 3429 m, Yungas cloud forest, 27 June 2010, A. Flakus 16855/2 & P. Rodriguez (KRAM; LPB).

###### Notes.

*Cora neoparabovei* is phylogenetically most closely related to *C. bovei*, *C. campestris*, *C. celestinoa*, *C. parabovei*, *C. palaeotropica*, *C. pseudobovei*, but morphologically and ecologically it is the most similar only to *C. parabovei* (Lücking et al. 2013, 2017). Both species are characterized by a rather unique pattern of dense hairs formed in concentric zones. *Cora neoparabovei*, is one of four described species with concentrically arranged surface hairs, aside from *C. dewisanti* B.Moncada, Suár.-Corr. & Lücking from Venezuela, Ecuador and Colombia, and *C. maxima* Wilk, Dal-Forno & Lücking and *C. parabovei* also from Bolivia. Nevertheless, *C. dewisanti* differs in mostly glabrous surface except for concentric bands of whitish setae, and with distinct, whitish, glabrous margins and presents a corticioid-cyphelloid hymenophore, *C. maxima* has glabrous lobe margins and is much larger, whereas *C. parabovei* has different colour of thallus when is dried (grey), which has concentric zonation both when fresh and dried (Lücking et al. 2017).

##### ﻿ Cora neopseudobovei

Taxon classificationFungiAgaricalesLichenomphaliaceae

Oset, Flakus & Guzow-Krzem. sp. nov.

39ACF797-F3AB-5FC5-91FF-9AD4E8D7B3B4

860020

Fig. 4

###### Diagnosis.

Species very similar to *C. pseudobovei*, but differing in the distinct phylogenetic position within the genus, and in substitution of several nucleotide posititions in nuITS (see also Suppl. material 2). The diagnostic features of the species are the small, macrosquamulose, up to 2 cm across brown upper surface thallus when fresh, pale yellow to orange-yellow when dry, with slightly visible concentric colour zonation when dry and also with involute, creamy white margins.

**Figure 4.**
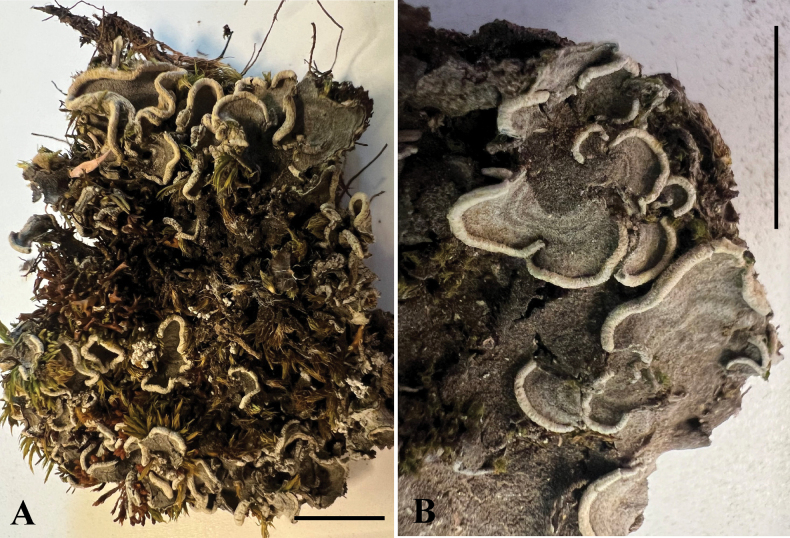
Morphology of *Cora neopseudobovei* (holotype). **A.** Upper surface (thallus is wet); **B.** Individual lobes (thallus is dry). Scale bars: 10 mm (**A, B**).

###### Type.

Bolivia • Dept. La Paz; Prov. Franz Tamayo, ANMIN Apolobamba, near Puyo Puyo village, 14 °56'55"S, 69°07'58"W, elev. 4888 m, high Andean open vegetation, 5 July 2010, A. Flakus 17603 & P. Rodriguez (holotype KRAM; isotype LPB).

###### Description.

Thallus lichenized, terricolous, foliose, small, macrosquamulose, up to 2 cm across, composed of up to 8 semicircular, adjacent to subimbricate lobes; individual lobes up to 1.2 cm wide and 6 mm long, moderately branched, without radial branching sutures, surface brown when fresh, with slightly visible concentric colour zonation when dry, with involute, creamy white margins. Upper surface shallowly undulate or scabrous when fresh and dry, involute margins undulate; lower surface excorticate, felty-arachnoid (representing the exposed medulla), brown-olive. Thallus in section 200–300 μm thick, with upper cortex, photobiont layer, and medulla; upper cortex collapsed compacted, formed by up 40 μm thick layer of loosely packed, irregularly arranged; photobiont layer up to 100 μm thick, orange above, aeruginous below, separated from the medulla by a thick, compacted layer of brownish hyphae; medulla 100 μm thick, often indistinct; clamp connections absent, papilliform hyphae absent. Hymenophore in section 50 μm thick, spores 5 × 3 μm, basidioles 20–25 × 3–6 μm. Sterigmata 4. Chemistry: No substances detected by TLC.

###### Habitat and distribution.

*Cora neopseudobovei* is known from four localities in La Paz department (Bautista and Franz Tamayo provinces), occurring at elevations between 3780 m and 4888 m in open area with shrubs and high Andean vegetation. The species was found on soil.

###### Etymology.

The name refers to the similarity in morphology to *Cora pseudobovei*.

###### Additional material examined.

Bolivia • Dept. La Paz; Prov. Bautista Saavedra, ANMIN Apolobamba, between la Curva and Charazani, 15°08'09"S, 69°02'03"W, elev. 3780 m, open area with shrubs, Ceja de Monte Superior (Altimontano), terricolous, 15 Nov. 2014, M. Kukwa 14700 (UGDA; LPB). • La Cumbre close to road Charazani-Pelechuco, 14°48'10"S, 69°10'51"W, elev. 4853 m, open high Andean vegetation close to lake and bofedales, 3 June 2017, A. Flakus 29538 (KRAM L-69723). • Prov. Franz Tamayo, ANMIN Apolobamba, Socondori Chico near Ulla Ulla village, 15°00'38"S, 69°13'48"W, elev. 4479 m, high Andean open vegetation, 4 July 2010, A. Flakus 17456 & P. Rodriguez (KRAM; LPB).

###### Notes.

All specimens of the new species form thalli of a similar size (up to 2 cm across, composed of 10–20 semicircular lobes) characterised by undulate upper surface, with smooth involute margins. The species is morphologically and ecologically similar to *C. bovei* and *C. pseudobovei* (Lücking et al. 2013, 2017). *Cora bovei* and *C. neopseudobovei* are characterised by undulate upper surface, but *C. bovei* forms larger lobes. While *C. pseudobovei* forms a thallus that is similar in size (up to 2 cm across, composed of 10–20 semicircular lobes), the involute margins are smooth, not undulate like in *C. neopseudobovei*, and the upper surface is darker (brown) than in the newly described species (Lücking et al. 2013, 2017).

According to Lücking et al. (2017) species forming the *Cora bovei* group are morphologically and ecologically similar to *C. squamiformis* Wilk, Lücking & Yánez-Ayabaca and *C. terricoleslia* Wilk, Dal-Forno & Lücking. However, on the global phylogeny of *Cora* they are in two unrelated clades, showing another striking example of parallelism in this genus (Lücking et al. 2017).

#### ﻿Species newly reported from Bolivia

##### ﻿ Cora arcabucana

Taxon classificationFungiAgaricalesLichenomphaliaceae

B. Moncada, C. Rodr, & Lücking, Fungal Diversity 84: 154 (2017)

1C3660B6-D226-5C02-9090-2DAA38442DB8

###### Description.

For the characteristics of the species, see Lücking et al. (2017).

###### Habitat and distribution.

Until now *Cora arcabucana* was noted from montane rain forest between 2,500 and 3,000 m of the northern Andes in Colombia (Lücking et al. 2017). The record of *C. arcabucana* given here is the first from Bolivia. Specimens were found at elevations between 2,250 m and 2,644 m in grazed and natural Yungas cloud forest.

###### Specimens examined.

Bolivia • Dept. Santa Cruz, Prov. Comarapa, PNANMI Amboró, Remate, 17°52'11"S, 64°20'53"W, elev. 2250 m, natural Yungas forest with big trees, 15 May 2017, P. Rodriguez 3885 (KRAM; LPB). • Prov. Manuel María Caballero, near Siberia, 17°50'38"S, 64°42'20"W, elev. 2644 m, grazed Yungas cloud forest with a large amount of bryophytes near road, 7 Nov. 2016, A. Flakus 28035 (KRAM; LPB).

###### Notes.

Both examined specimens of *Cora arcabucana* were characterised by aeruginous-green lobes without concentric colour zonation, with thin, involute, greenish-grey margins when fresh, with scattered surface hairs and scattered, dark, marginal soredia, as well as an adnate-confluent and emarginate hymenophore.

*Cora arcabucana* is morphologically related to *C. davidia* B.Moncada, L.Y.Vargas & Lücking with which it shares the epiphytic growth habit and the adnate-confluent hymenophore, as well as the numerous papillae developed on the lower surface (see Lücking et al. 2014c). According to Lücking et al. (2017)*C. davidia* is a medium-sized, epiphytic species characterised by marginal soredia, with adnate confluent, emarginate hymenophore; meanwhile thallus of *C. arcabucana* produces sparse soredia, the surface is thinly pilose, and the papillae are narrower and more branched; in addition, the habitat ecology is different, with *C. davidia* being known only from paramo whereas *C. arcabucana* is a montane forest species. Another similar, closely related species is *C. garagoa* Simijaca, B.Moncada & Lücking, which is slightly larger than *C. arcabucana* and lacks soredia. Nevertheless, they are phylogenetically and ecologically different (Lücking et al. 2017 and this study).

According to Lücking et al. (2017) both *Cora arcabucana* and the other mentioned species belong to the *C. byssoidea* clade represented by e.g., *C. byssoidea* Lücking & B. Moncada, *C. dalehana* B. Moncada, Madriñán & Lücking, *C. putumayensis* L.J.Arias, B. Moncada & Lücking, *C. davibogotana* Lücking, B. Moncada & Coca, and others,which is a group of species with a hymenophore forming rounded, adnate patches resembling the ascomata of the lichenized genus *Myriostigma*.

##### ﻿ Cora cuzcoensis

Taxon classificationFungiAgaricalesLichenomphaliaceae

Holgado, Rivas Plata & Perlmutter, Fungal Diversity 84: 166 (2017)

0902A02A-F0E9-5078-9DE7-2B4A7695E9C5

###### Description.

For the characteristics of the species, see Lücking et al. (2017).

###### Habitat and distribution.

Until now *Cora cuzcoensis* was only known from the type locality near Machu Picchu in Peru, growing on the ground in the highly disturbed subandine rain forest, at an altitude of 3,500 m (Lücking et al. 2017). The record of *C. cuzcoensis* given here is the first from Bolivia. Specimens were found at elevation 3545 m in open anthropogenic area. The specimen was found on soil.

###### Specimens examined.

Bolivia • Dept. La Paz; Prov. La Paz, Prov. Larecaja, Jocollone village, Paramo Yungeño, 15°37'35"S, 68°41'21"W, elev. 3545 m, open anthropogenic area, on terricolous, 14 May 2011, A. Flakus 20419 & O. Plata (KRAM L-66937; LPB).

###### Notes.

According to Lücking et al. (2017)*Cora cuzcoensis* is a terrestrial species characterised by rather dark, olive-green lobes. Meanwhile, the thallus of the examined specimen was dark in the center and grey to greyish-white at the edge of the thallus. The most similar species is *C. caliginosa* Holgado, Rivas Plata & Perlmutter from the same habitat, which differs in the dark olive-grey lobes without greenish tinge and the thicker thallus. Both species are not phylogenetically closely related (Lücking et al. 2017).

##### ﻿ Cora undulata

Taxon classificationFungiAgaricalesLichenomphaliaceae

L. Vargas, Moncada & Lücking, The Bryologist 117 (4): 374 (2014)

7B29435D-D525-5226-8509-B74CC4536EA9

###### Description.

For the characteristic of the species, see Vargas et al. (2014).

###### Habitat and distribution.

Until now *Cora undulata* was only known from the type locality in Colombia growing on rocks and associated with bryophytes in more or less exposed microhabitats (Vargas et al. 2014). The record of *C. undulata* given here is the first from Bolivia. Specimens were found at an elevation of 544 m in the Sub-Andean Yungas forest.

###### Specimens examined.

Bolivia • Dept. Cochabamba; Prov. Chapare, Parque Nacional Carrasco, Camino de las Nubes near Guacharos, 17°05'06"S, 65°29'12"W, elev. 544 m, Sub-Andean Yungas forest, July 2009, P. Rodriguez 3621 (KRAM; LPB).

###### Notes.

The examined specimen of *Cora undulata* has foliose thallus smaller (up to 7.5 cm diam.) than specimens from Colombia (up to 9 cm diam.). Upper surface is dark olive-green when fresh, but when dried greyish white, with fine but distinct concentric ridges, glabrous. The species is morphologically close to *C. ciferrii* (Tomas.) Lücking, A. Grall & Thüs. It is characterised by an undulated surface and the hymenophore forming larger, more irregular patches that often become confluent (Lücking et al. 2014a). In comparison, *C. undulata* forms smaller lobes with dark olive-green colour when fresh and is saxicolous (Vargas et al. 2014).

#### ﻿New localities from Bolivia

##### ﻿ Cora aspera

Taxon classificationFungiAgaricalesLichenomphaliaceae

Wilk, Lücking & E. Morales, Phytotaxa 139 (1): 8 (2013)

5F4111D9-ED98-5EE6-ADCB-A934823C8578

###### Description.

For the characteristics of the species, see Lücking et al. (2013).

###### Habitat and distribution.

*Cora aspera* is known from several collections from Costa Rica, Colombia, Ecuador, Bolivia, and Peru. According to Lücking et al. (2013), it appears to be a primarily epiphytic species, growing on twigs and branches of trees and shrubs in montane rain forest and paramo vegetation.

The species was recorded in Bolivia several times (Lücking et al. 2013; Flakus et al. 2014), however, only three records presented by Lücking et al. (2013), were confirmed by nuITS rDNA sequences. The unsequenced records presented by Flakus et al. (2014) may belong to other species.

###### Specimens examined.

Bolivia • Dept. Santa Cruz; Prov. Caballero, Siberia region near La Palma, 17°49'12"S, 64°40'28"W, elev. 2582 m, the Yungas cloud forest, on trunk among liverworts, 13 Dec. 2004, A. Flakus 4705 (KRAM L-49626; LPB). • Prov. Manuel María Caballero, near Siberia, 17°50'38"S, 64°42'20"W, elev. 2644 m, grazed Yungas cloud forest with a large amount of bryophytes near road, 7 Nov. 2016, A. Flakus 28025 (KRAM; LPB). • Monte Empalme near Siberia, 17°50'05"S, 64°42'09"W, elev. 2439 m, partly grazed Yungas cloud forest near stream, 8 Nov. 2016, P. Rodriguez 3579 (KRAM; LPB). • Dept. Santa Cruz, Prov. Manuel María Caballero, East Cordillera, Siberia region near La Palma village, 17°49'12"S, 64°40'28"W, elev. 2582 m, Yungas cloud forest, 12 Dec. 2004, A. Flakus 4591 (KRAM; LPB). • Prov. Franz Tamayo, PNANMI Madidi, below Keara Bajo, 14°41'47"S, 69°04'10"W, elev. 3160 m, open area with shrubs and scattered trees, 18 Nov. 2014, A. Flakus 25280 (KRAM; LPB).

###### Notes.

*Cora aspera* is one of the several species growing typically as an epiphyte, and it is the largest and most common epiphytic species known in the genus. Together with *C. boleslia* Lücking, E. Morales & Dal-Forno, *C. canari* Nugra, Dal-Forno & Lücking, *C. corani* Lücking, E. Morales & Dal-Forno, *C. dulcis* Moncada, Pérez-Pérez & Lücking, *C. fimbriata* L. Vargas, Moncada & Lücking, and others, it makes up the *C. aspera* clade, which includes mostly epiphytic taxa with more or less green thallus when fresh, frequently forming medullary papillae and growing mostly in montane forest (Lücking et al. 2017).

All examined specimens were characterised by foliose thallus, up to 9 cm across (bigger than previously noted) composed of 2–5 semicircular lobes per thallus; lobes 2–5 cm wide and 1–5.5 cm long, often branched and with short radial branching sutures, pale greenish grey with slight concentric color zonation when fresh, and with thin but distinct, involute, white to pale grey margins, becoming white to grey when dried.

## Supplementary Material

XML Treatment for Cora neoparabovei

XML Treatment for Cora neopseudobovei

XML Treatment for Cora arcabucana

XML Treatment for Cora cuzcoensis

XML Treatment for Cora undulata

XML Treatment for Cora aspera

## ﻿Additional information

### Use of AI

No use of AI was reported.

### Author ORCIDs

Magdalena Oset https://orcid.org/0000-0003-4068-434X

Adam Flakus https://orcid.org/0000-0002-0712-0529

Beata Guzow-Krzemińska https://orcid.org/0000-0003-0805-7987

### Data availability

All of the data that support the findings of this study are available in the main text or Supplementary Information.

## Supplementary materials

Supplementary material 1 Specimens of *Cora* used in molecular analysis Data type xlsx Explanation note Specimens of *Cora* used in molecular analysis with GenBank accession numbers, voucher numbers, country of origin, and source publication. Sequences generated for this study are in bold. https://binary.pensoft.net/file/1482070This dataset is made available under the Open Database License (http://opendatacommons.org/licenses/odbl/1.0/). The Open Database License (ODbL) is a license agreement intended to allow users to freely share, modify, and use this Dataset while maintaining this same freedom for others, provided that the original source and author(s) are credited.Magdalena Oset, Adam Flakus, Beata Guzow-Krzemińska

Supplementary material 2 Variable positions in the alignment of nuITS rDNA marker Data type xlsx Explanation note Variable positions in the alignment of nuITS rDNA marker of *Cora bovei*, *C. neoparabovei*, *C. neopseudobovei*, *C. palaeotropica*, *C. parabovei*, *C. pseudobovei* and *Cora* sp. Variable characters are marked in bold, while diagnostic nucleotide position characters to distinguish these species from other taxa of this group are marked with a yellow background. https://binary.pensoft.net/file/1482071This dataset is made available under the Open Database License (http://opendatacommons.org/licenses/odbl/1.0/). The Open Database License (ODbL) is a license agreement intended to allow users to freely share, modify, and use this Dataset while maintaining this same freedom for others, provided that the original source and author(s) are credited.Magdalena Oset, Adam Flakus, Beata Guzow-Krzemińska
